# Supplementary material for: EEG-Pype: An accessible MNE-Python pipeline with graphical user interface for preprocessing and analysis of resting-state electroencephalography data
Source: PLoS Comput Biol. 2026 Mar 2;22(3):e1014043. doi: 10.1371/journal.pcbi.1014043 (PMC12970966; doi:10.1371/journal.pcbi.1014043)
Supplement: Attachment — Submitted filename: review.docx [file pcbi.1014043.s002.docx]

The paper describes an open source graphical user interface application “EEG-Pype” that uses MNE-Python functions. The application designed for smooth defined processing of resting state EEG data to use for researchers without programming experience.
The work demonstrates a functioning pipeline; however, it does not appear to introduce novel elements beyond what is already available in the literature. Its current rigidity (only resting state data) and the omission of basic functionality (e.g., triggers) constrain its practical utility. The tool may nonetheless provide value in narrow contexts. Intuitive graphical user interface removes some barriers for researchers to start processing their data, which is great for the community. And as the GitHub page with application is public the interface has a potential to grow if community needs more.

Major concerns:
1) My main concern is the installation instructions. Since “EEG-Pype” is designed for researchers without much technical experience, the current guide doesn’t feel very user-friendly for its target audience. I found myself struggling with the installation process as well.

Would it be possible to add another video demonstrating the full installation workflow, or include additional screenshots for each step? I think this would make the process much clearer for new users

2) The instructions on GitHub for the scrips have couple of inaccuracies:
 a) in the section “Quantitative analysis module (separate script)”
the script is not in the folder “/EEG-Pype”, but in the folder “EEG-Pype/scr”

b) I would add where the test data in the GitHub. In the paper L238 mentions “a BioSemi.bdf file” as a tested data. However, the data name at the GitHub “EEG-Pype_test_EEG.bdf”

Minor concerns:

1) L151 – What is the default number of ICA components?
2) L170 Please explain the choice of regularization parameter?
3) Is it possible to save the figures? Not just data?
4) L243 please add the link to the GitHub page.

Suggestion:

Being able to extract the raw Python mne-python script generated through the interface could be very beneficial—both for collaboration and for making custom modifications to the processing pipeline.
