# Supplementary material for: EEG-Pype: An accessible MNE-Python pipeline with graphical user interface for preprocessing and analysis of resting-state electroencephalography data
Source: PLoS Comput Biol. 2026 Mar 2;22(3):e1014043. doi: 10.1371/journal.pcbi.1014043 (PMC12970966; doi:10.1371/journal.pcbi.1014043)
Supplement: Attachment — Submitted filename: Response_to_Reviewers.docx [file pcbi.1014043.s004.docx]

**Response to Reviewers**

**Manuscript ID:** PCOMPBIOL-D-25-02246

**Title:** EEG-Pype: an accessible MNE-Python pipeline with graphical user interface for preprocessing and analysis of resting-state electroencephalography data

Dear Editors and Reviewers,

We thank the editors and reviewers for their constructive comments and suggestions. We have carefully addressed all points raised and believe the manuscript has been substantially improved as a result. Below, we provide detailed point-by-point responses to each comment. Reviewer comments are shown in *italics*, and our responses follow in regular text.

───────────────────────────────────────

**EDITOR COMMENTS**

*The authors should ensure that the procedure for installing the GUI is made as straightforward as possible, and that the other support documentation is clear and error free, to maximise the potential impact of this software.*

**Response:** We thank the editor for this guidance. We have substantially expanded the installation instructions on our GitHub page to include more detailed steps and screenshots for users with less technical background. Additionally, we have corrected inaccuracies in the documentation (as detailed in our responses to Reviewer 1) and reorganized the support materials to improve clarity. We believe these changes will significantly lower the barrier to entry for our target audience.

**REVIEWER 1**

*The paper describes an open source graphical user interface application "EEG-Pype" that uses MNE-Python functions. The application designed for smooth defined processing of resting state EEG data to use for researchers without programming experience. The work demonstrates a functioning pipeline; however, it does not appear to introduce novel elements beyond what is already available in the literature. Its current rigidity (only resting state data) and the omission of basic functionality (e.g., triggers) constrain its practical utility. The tool may nonetheless provide value in narrow contexts. Intuitive graphical user interface removes some barriers for researchers to start processing their data, which is great for the community. And as the GitHub page with application is public the interface has a potential to grow if community needs more.*

**Major Concerns**

*1) My main concern is the installation instructions. Since "EEG-Pype" is designed for researchers without much technical experience, the current guide doesn't feel very user-friendly for its target audience. I found myself struggling with the installation process as well. Would it be possible to add another video demonstrating the full installation workflow, or include additional screenshots for each step? I think this would make the process much clearer for new users.*

**Response:** We would like to thank the reviewer for raising this point; and we agree. Therefore, we have expanded the installation instructions in the README on our GitHub page to include more details for users with less of a technical background. In addition, we have included screenshots in this section to further clarify the installation.

*2) The instructions on GitHub for the scripts have a couple of inaccuracies:*

*a) In the section "Quantitative analysis module (separate script)" the script is not in the folder "/EEG-Pype", but in the folder "EEG-Pype/src"*

*b) I would add where the test data is in the GitHub. In the paper L238 mentions "a BioSemi.bdf file" as tested data. However, the data name at the GitHub is "EEG-Pype_test_EEG.bdf"*

**Response:** We agree that these two points created unnecessary confusion and have addressed both, changing the folder path in the GitHub README and modifying the referenced sentence to:

"To demonstrate the functionality and output of the EEG-Pype workflow, we processed the sample 64-channel resting-state EEG measurement provided with the software (a BioSemi .BDF file: EEG-Pype_test_EEG.bdf, located in the 'EEG-Pype/src' folder once EEG-Pype is installed)."

**Minor Concerns**

*1) L151 – What is the default number of ICA components?*

**Response:** We have added this information to this sentence:

"A custom number of total ICA components can be chosen, influencing the number of components the signal is decomposed to (default: 25 components)."

*2) L170 Please explain the choice of regularization parameter?*

**Response:** We thank the reviewer for this question. The regularization parameter λ=0.05 was selected based on the standard implementation defaults in MNE-Python (Gramfort et al., 2013). This parameter controls Tikhonov regularization (diagonal loading) by adding 5% of the average sensor variance to the diagonal of the covariance matrix before inversion.

This step is important for EEG data, particularly after average referencing, as the data covariance matrix becomes rank-deficient. Regularization ensures the matrix is invertible and stabilizes the beamformer weights against numerical instability and noise. We have updated the manuscript to clarify that this parameter represents 5% of the average sensor variance. The expanded section now reads:

"Tikhonov regularization is applied (λ=0.05). This adds 5% of the mean sensor variance to the diagonal of the covariance matrix, ensuring numerical stability while preserving spatial resolution (3)."

*3) Is it possible to save the figures? Not just data?*

**Response:** We do not, at this moment, have the option to automatically save images (such as the PSD plots) to a file. There is however a very easy way to do this in the MNE-Python interface of the plot, by pressing the save icon. We have clarified this as follows (L266):

*“Upon loading the raw .BDF file, EEG-Pype first presents an unfiltered PSD plot. This initial visualization allows the user to assess the overall data quality and identify prominent artifacts, such as line noise. This plot can be saved using the built-in MNE-Python save functionality by pressing the save icon.”*

*4) L243 please add the link to the GitHub page.*

**Response:** We have added the link in the suggested location.

**Suggestion**

*Being able to extract the raw Python mne-python script generated through the interface could be very beneficial—both for collaboration and for making custom modifications to the processing pipeline.*

**Response:** We thank the reviewer for this suggestion and agree that facilitating collaboration and allowing for custom modifications are essential features for open-source tools. We have addressed this requirement through two core design choices in EEG-Pype:

**Reproducibility via Configuration Files:** Instead of generating a new script for every run, EEG-Pype saves a comprehensive configuration file (.pkl) alongside the processing logs. This file captures every parameter setting, channel selection, and epoch selection used in a session. Collaborators can load this file to exactly reproduce a processing pipeline on their own machines without needing to manually replicate settings or write code.

**Open-Source Modifiability:** Unlike compiled GUI tools (e.g., legacy Java executables), EEG-Pype is distributed as raw Python source code. Advanced users wishing to make custom modifications can directly edit the source Python script (specifically the function definitions provided in the repository) rather than needing an exported intermediate script.

We believe this approach provides the best of both worlds: a user-friendly GUI for standard use, and direct access to the source code for advanced users who wish to fork or modify the pipeline logic. We have clarified this in the "Design and Implementation" section of the manuscript.

───────────────────────────────────────

**REVIEWER 2**

*The authors developed an interactive GUI that wraps up MNE-Python functions to provide: a) preprocessing and b) analysis pipelines for rsEEG signals. Overall, the authors aim to simplify access to standardized methods for rsEEG processing among researchers without programming experience. The latter could be particularly helpful in paving the way for clinicians involved in neuroscience research. This reviewer salutes the initiative and considers the manuscript worthy of publication with minor changes. Similarly, minor changes are suggested in the pipeline implementation, accounting for user experience and potential needs for research applications.*

**Minor Recommendations for the Manuscript**

*1) On page 9 (lines 126 & 127), please consider pointing the reader to the specific function and .py script for expanding the read raw functions.*

**Response:** Considering the addition of the new mne.io.read_raw function (see our response to comments A1-3), this addition is no longer relevant.

*2) On page 9 (lines 143 & 144), please state in the manuscript the selected frequencies for the bandpass filter prior to ICA (1 – 47 Hz).*

**Response:** We have included this detail in the manuscript:

"ICA calculation is based on a bandpass filtered (1.0-47.0 Hz) copy of the EEG"

*3) On page 9 (lines 143 & 144), please direct the readers to the perform_ica() function, as some users would like to modify ICA parameters or method beyond the number of components.*

**Response:** We have included this information in the manuscript as follows:

"Independent component analysis (ICA) can be performed using the standard MNE-Python implementation (3). This functionality is centered around the perform_ica() function in the EEG-Pype source code."

*4) On page 12 (lines 210 & 211), please state that entropy-based features are not calculated across the channel time series (broadband) but over a filtered version for each frequency band (returning the entropy by band based on previously reported methods by the authors). Although this clarification can be inferred from the tables, declaring it will provide a clearer methodology for the reader.*

**Response:** We have included this information in the first paragraph of this section as follows:

"EEG-Pype can be used to calculate several quantitative EEG measures after preprocessed EEG data is saved. Power Spectral Density (PSD) measures are calculated on the epochs saved in the broadband, while entropy, functional connectivity and by extension topology measures are calculated on all previously exported frequency bands."

**Recommendations for the GUI & Pipeline Implementation**

**A) Preprocessing module – Set montage & Read raw files**

*1) In the "Enter filetype" window, consider adding a "More Info" button leading to the MNE "work with sensor data" tutorial, or ideally, consider adding some images generated with the plot_sensors() so the user can "preview" the expected ch_names and inspect if the montage is appropriate for the data.*

*2) Also consider expanding (or pointing the author to the set_montage function) to the 10-10 and 10-05 montages, which can be suitable for datasets with a high density channel number.*

*3) It would be great to set mne.io.read_raw() as the default reading function (as it wraps over much more file formats than the currently available).*

**Response:** We thank the reviewer for their suggestions. It is really helpful that they took the time to provide us with these in-depth comments, and we have implemented all three of the suggestions above. We moved to a more robust raw EEG file reading system based on mne.io.read_raw(), expanding the formats EEG-Pype is able to recognize. In the process, we also streamlined the montage selection and added additional info and a 10-05 montage (10-10 is not available in MNE but 10-05 should work fine for this use case as well). This change is reflected in the manuscript as well:

"By using MNE-Python's generic I/O interface, EEG-Pype supports the automatic detection and import of a broad range of standard electrophysiological formats (e.g., BDF, EDF, FIF, BrainVision, CNT, EEGLAB, EGI). Additionally, a custom parser is included to handle plain ASCII (*.txt) files with automatic delimiter detection and configurable header parsing."

*4) Following the above comment, if the authors want to keep the individual reader functions, please state in the manuscript that ".cnt" supported files come from NeuroScan amplifiers, not ANT Neuro (which require read_raw_ant()).*

**Response:** This suggestion is no longer relevant considering the above change.

**B) Preprocessing module – Set Ch_names**

*1) If ch_names doesn't conform to the expected names (due to suffixes like "-AVG" or prefixes like "EEG"), it would be nice to add a destrip function to run over all channels with suffixes or prefixes. More importantly, authors can consider applying the standardize() function (it changes the midline "Z" suffix to lowercase, the latter is required for some devices like NeuroScan).*

**Response:** We thank the reviewer for this suggestion regarding channel name standardization. We agree that inconsistencies in channel naming conventions (such as suffixes like "-AVG", prefixes like "EEG ", or the uppercase/lowercase midline "Z" issue) are a common source of problems in EEG preprocessing workflows.

We would like to highlight that the current version of EEG-Pype already includes functionality to address these issues through the Channel Name Correction window, which features a "Find and Replace" tool. This allows users to quickly strip prefixes or suffixes (e.g., replacing "EEG " with "" or "-AVG" with "") and apply case corrections across all channel names in a single operation. We opted for this semi-automated approach because it keeps the user informed of exactly what transformations are applied to their data, which we believe is important for transparency and reproducibility. Additionally, it accommodates the wide variety of naming conventions encountered across different acquisition systems and research contexts, some of which may require non-standard handling.

We acknowledge that incorporating automatic standardization (such as MNE's standardize() function) as an optional preprocessing step could further streamline the workflow for common cases. We will consider adding this as an optional feature in a future release, while retaining the current manual correction option for users who want more fine-grained control.

*2) It may also be useful to support inheriting the "corrected" ch_names from one EEG file to subsequent files, so the user doesn't have to repeat the correction for every file when channel names are consistent across recordings.*

**Response:** We appreciate this suggestion, as repeating channel name corrections for each file can indeed be tedious when recordings share consistent naming conventions.

An earlier version of EEG-Pype did include functionality to inherit channel name corrections across all files in a batch. However, we found that this approach introduced issues when users processed batches containing files with (often unnoticed) differences in channel names or channel counts. These inconsistencies would cause batch processing to fail, leading to frustration. Therefore, we chose to handle channel name corrections on a per-file basis. While this requires more user interaction, it ensures that each file is explicitly verified before processing, reducing the risk of silent errors or batch failures. For users with homogeneous datasets, the "Find and Replace" functionality allows for rapid corrections that typically require only a few keystrokes per file.

**C) Preprocessing module – Set montage, ICA & Filters**

*1) Consider allowing the user to reassign ch_names if mistakes/typos happen. In my use case, I edited "F8-AVG" and wrote "F7" then it was not possible to reassign it or repair the mistake.*

*2) Similarly, it would be advisable to assert that there are no duplicate ch_names before running the preprocessing pipeline for a given file.*

**Response:** These are, again, very sensible additions and we have implemented them by adding an "undo last change" option and "reset to original" option to our channel correction window. At the same time, EEG-Pype now checks for duplicate channel names right in the same window when applying channel name corrections. These additions should prevent the situation you describe from happening to users.

*3) For non-experienced researchers assessing spectral and topographical patterns of brain-like and non-brain-like Independent Components, it would be advantageous to incorporate MNE ICALabel into the pipeline, returning the predicted label for each IC, as an optional tick check, so the user can also get assistance in the IC selection.*

**Response:** Thanks again for this suggestion. We have incorporated ICALabel as an optional feature (checkbox in the GUI) so that the user can see the estimated labels per component concurrently with the topographical and time series ICA plots. The user is still asked to make a selection, but they can cross-reference the different information to do this. We have included this addition in the manuscript as follows:

"To aid in selecting possibly artifactual ICA components, three plots are shown: head plots showing the spatial distribution of the components, temporal plots showing the time series of each component across the entire measurement (Figure 2) and optionally a list of components with an estimated source (i.e, brain, eye, muscle, heart, line noise, channel noise or other) and confidence percentages based on MNE-Python's ICALabel functionality.”

At the same time, we have revised the ICA part of the Results section slightly to also reflect this new functionality, including a new Figure 2.

**D) Analysis module**

*1) From the user perspective, I would recommend rearranging the spatial position of each box (Input Settings, Matrix Export, Complexity Measures…) so that the qEEG analysis GUI resembles each of the steps of the pipeline.*

**Response:** We have now rearranged the different blocks in the Quantitative Analysis Module GUI in three steps: Step 1: Setup (input settings and number of epochs analyzed), Step 2: Analysis (different analysis options), Step 3: Output and Run (Matrix Export options and Log Output). This aligns the layout more with the underlying process.

*2) Also, consider if JPE should be placed in the Connectivity box.*

**Response:** We have actually thought about this arrangement before, but because JPE builds on PE and they share the time step (tau) setting, we feel this would create unnecessary confusion. Therefore, we have decided to leave the arrangement as is for now.
